# TECHNIQUES FOR OVERCOMING CHALLENGES TO RESEARCH WITH INDIVIDUALS NEARING THE END OF LIFE

**DOI:** 10.1093/geroni/igad104.1311

**Published:** 2023-12-21

**Authors:** Ellis Dillon, Meghan Martinez, Martina Li, Alyssa Hernandez, Su-Ying Liang, Manali Patel

**Affiliations:** UConn Health, Farmington, Connecticut, United States; Sutter Health, Palo Alto, California, United States; Sutter Health, Palo Alto, California, United States; Sutter Health, Palo Alto, California, United States; Sutter Health, Palo Alto, California, United States; Stanford University, Stanford, California, United States

## Abstract

People living with advanced disease and nearing the end of their lives are often omitted in research on healthcare and the lifespan. This omission occurs for practical, ethical, and cultural reasons. Drawing on examples from studies on individuals receiving home hospice care, and individuals with advanced cancer and their caregivers, we will review (1) practical, ethical, and cultural challenges to doing this research, (2) techniques to increase access, recruitment, and participation through developing partnerships, flexibility and resource allocation, and (3) research team training and support. Practical challenges include the unpredictability of illness and symptoms leading to cancellations and increased attrition, and increased time and resources needed to recruit, consent, and travel to community residences. Ethical and cultural challenges include concerns about “vulnerability,” and stigma around directly discussing dying. Techniques to facilitate successful inclusion of these populations include developing partnerships with community organizations, clinical teams, and patient or family advocates, justifying and allocating sufficient resources and flexibility in study design and word choices to reflect the spectrum of understanding and experiences, and additional training and support for research staff. Other techniques to include this population include use of secondary data, reconsidering research exclusion criteria, and surveying/interviewing family members and other caregivers. Hard to reach populations are often overlooked, yet they are vital to ensuring full representation of lived experience. Science benefits from developing strategies to include these populations in research.

